# Blood to the Bone: Epistaxis Leading to Osteomyelitis

**DOI:** 10.1155/carm/3934087

**Published:** 2025-09-30

**Authors:** Zarif Kazi, Bettina Zou, Marlon Rivera Boadla, Edward Chapnick

**Affiliations:** Maimonides Medical Center, Brooklyn, New York, USA

**Keywords:** apixaban, atrial fibrillation, epistaxis, osteomyelitis

## Abstract

Apixaban is a direct Factor Xa inhibitor that has been increasingly prescribed, particularly for stroke prevention in atrial fibrillation. While safer than warfarin, it still carries bleeding risks, including epistaxis, which is commonly managed with nasal packing. We report a 75-year-old woman on apixaban who developed methicillin-resistant *Staphylococcus aureus* (MRSA) bacteremia after nasal packing for epistaxis. She presented a few weeks later with worsening back pain and was diagnosed with L2-L3 vertebral osteomyelitis from MRSA. The case highlights the rare risk of distant osteomyelitis following nasal packing and the importance of evaluating changes in chronic pain.

## 1. Background

Apixaban is a direct Factor Xa inhibitor primarily used for stroke prevention in nonvalvular atrial fibrillation as well as for treatment and prevention of deep vein thrombosis and pulmonary embolism. The preference by clinicians for direct oral anticoagulants (DOACs) over warfarin has increased over the past decade [1]. By 2013, DOACs were prescribed more frequently than warfarin, and apixaban was the most common DOAC prescribed [2]. While DOACs are associated with a lower risk of bleeding than warfarin [3, 4], the risk of bleeding from DOACs should still be considered.

The rate of nonmajor bleeding in patients taking apixaban was found to be 6.4 per 100 patient-years, and epistaxis accounted for 14.8% of all nonmajor bleeding events [5]. Nasal packing is a common treatment for epistaxis and is associated with several well-documented complications including infections, septal hematomas and abscesses, pressure necrosis, neurogenic syncope, trigeminocardiac reflex and aspiration of packing material [6–8]. There are limited studies currently describing the relationship between nasal packing and osteomyelitis. In this article, we present the case of an elderly patient who suffered a major episode of epistaxis complicated by bacteremia and remote lumbar osteomyelitis.

## 2. Case Report

**Figure 1:**
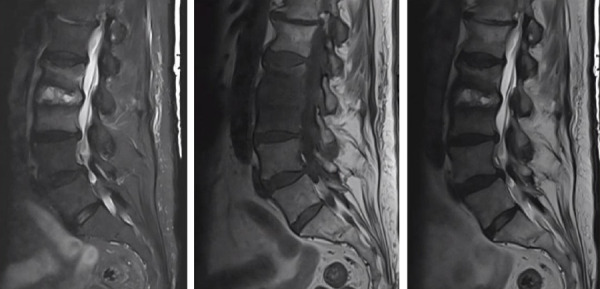
MRI of the lumbar spine which shows infiltrative T1 marrow signal involving L2 and L3 vertebral bodies with associated postcontrast enhancement. Fluid/edema within the L2-L3 disc space.

A woman in her 70s with atrial fibrillation being treated with apixaban, and chronic back pain due to spinal stenosis presented from a skilled nursing facility with 3 weeks of worsening back pain. She was recently discharged from another hospital where she had presented with major epistaxis requiring nasal packing and multiple units of red cell transfusion. The patient had anterior nasal packing for about 48 h with no concurrent antibiotics. It was unclear which type of nasal packing was used. The hospital course was complicated by methicillin-resistant *Staphylococcus aureus* (MRSA) bacteremia secondary to nasal packing, which was treated with intravenous vancomycin for 6 weeks. The patient was discharged 4 weeks prior and completed antibiotics 2 weeks prior to this admission. Placement of a left atrial appendage (LAA) closure device was planned, as anticoagulation had been discontinued because of the significant bleeding. An MRI, as shown in Figure 1, showed evidence of discitis and osteomyelitis at L2-L3, and culture of lumbar disc sampling showed MRSA. The patient had developed vertebral osteomyelitis 3 months after nasal packing was initially placed. Of note, blood cultures showed no growth. Treatment with daptomycin was started. Placement of the LAA closure device was postponed due to ongoing infection.

**Figure 2:**
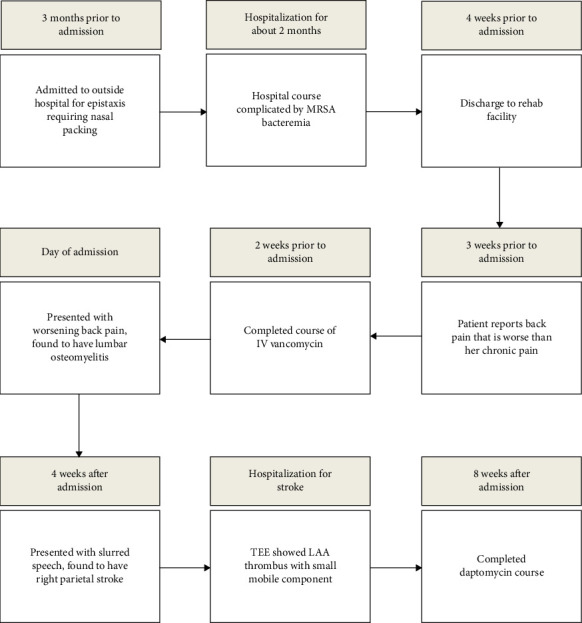
Patient's clinical course and timeline including prior hospitalization and postdischarge complications.

The patient returned to the hospital from the rehabilitation facility 4 weeks later due to episodic slurred speech and confusion for one day. On physical examination at the time of presentation, the only neurologic deficit was difficulty recalling words at times. CT scan of the brain showed no abnormal acute intracranial findings and mild chronic microvascular disease. MRI of the brain showed a punctate focus of restricted diffusion in the right parietal lobe, consistent with an acute/subacute infarct. Transesophageal echocardiogram (TEE) showed a LAA thrombus measuring 2.6 cm × 1.3 cm with a small mobile component. Daptomycin was continued to complete 8 weeks of therapy.  Figure 2 depicts the patient's clinical course from initial hospitalization due to epistaxis to the most recent admission for stroke.

## 3. Discussion

The use of DOACs, such as apixaban, has increased significantly over the past 2 decades. Between 2013 and 2018, apixaban prescriptions in the United States increased from 75,948 to 7,741,247 among Medicare beneficiaries [1]. Epistaxis is a known complication in patients on anticoagulation therapy, with nasal packing being a common intervention. In the United States, 19.7% of Emergency Department visits for epistaxis require nasal packing to control [9]. However, nasal packing can predispose patients to secondary infections such as bacteremia [10]. The moist and enclosed environment created by the nasal packing can serve as a nidus for bacterial growth. *S. aureus* has been known to colonize several parts of the body, including the anterior nares [11].

In our patient, it is likely that the nasal packing contributed to the development of MRSA bacteremia. The subsequent development of osteomyelitis in the lumbar spine (L2-L3) highlights a severe and rare complication of bacteremia. This case highlights the need for careful monitoring and management of patients on anticoagulation therapy who develop epistaxis. As remote osteomyelitis is a rare complication of nasal interventions, there is currently insufficient epidemiological data. However, there are reports such as right first rib costal osteomyelitis in a patient after nasal septoplasty [12]. Currently, the American Academy of Otolaryngology—Head and Neck Surgery does not recommend antibiotic prophylaxis for nasal packing. Although rare, physicians must be vigilant for symptoms of osteomyelitis in patients with known risk factors, such as those with increased risk of bleeding in patients on anticoagulation.

Osteomyelitis can present in various anatomical locations, each with distinct clinical manifestations. In the vertebral column, it typically presents with localized back pain, tenderness, and sometimes fever or neurological deficits due to spinal cord compression [13]. Vertebral osteomyelitis is often a result of hematogenous spread from remote sites such as the skin, urinary tract, and invasive lines; however, as seen in this case, it can originate from other distant sites as well. In our patient, the nasal packing was either the source of bacteria being introduced into the nasal mucosa or provided a nidus for growth. Once the bacteria entered the bloodstream, the circulating bacteria seeded into bone tissue. This occurs particularly in areas that have a rich vascular supply such as the metaphyseal areas of long bones or vertebrae. Once in the bone, the bacteria adhere to the endothelium followed by extravasation into the bone marrow, where they proliferate. This triggers an inflammatory response, which leads to tissue destruction and necrosis [14].

It is important for physicians to pay importance to a change in pain symptoms in patients with chronic pain. Our patient had been reporting worsening back pain for 3 weeks but it is likely that her complaints were initially attributed to her chronic pain secondary to spinal stenosis. This likely delayed the patient's diagnosis by a few weeks. Early identification is crucial as highlighted by this case. The patient was off anticoagulation and pending an LAA closure device. The delay in diagnosis and subsequent treatment meant she was off anticoagulation for longer, leading to the formation of a LAA thrombus and subsequent stroke.

Although rare, nasal packing can lead to severe systemic infections, including MRSA bacteremia and osteomyelitis. A thorough history of recent interventions, including nasal packing, can provide important diagnostic clues. Physicians should remain vigilant for atypical presentations of infection following nasal packing, particularly in high-risk patients such as those on anticoagulation therapy. Close follow-up and prompt recognition of systemic signs of infection can prevent severe complications, emphasizing the importance of an interdisciplinary approach in managing these patients.
